# S-Adenosyl-L-Methionine and Cu(II) Impact Green Plant Regeneration Efficiency

**DOI:** 10.3390/cells11172700

**Published:** 2022-08-30

**Authors:** Renata Orłowska, Jacek Zebrowski, Janusz Zimny, Piotr Androsiuk, Piotr Tomasz Bednarek

**Affiliations:** 1 Plant Breeding and Acclimatization Institute—National Research Institute, Radzików, 05-870 Błonie, Poland; 2 Institute of Biology and Biotechnology, University of Rzeszow, Pigonia 1, 35-310 Rzeszow, Poland; 3 Department of Plant Physiology, Genetics and Biotechnology, Faculty of Biology and Biotechnology, University of Warmia and Mazury in Olsztyn, 10-719 Olsztyn, Poland

**Keywords:** anther culture, copper, genetic variation, metAFLP, S-adenosyl-L-methionine, silver, triticale

## Abstract

The biological improvement of triticale, a cereal of increasing importance in agriculture, may be accelerated via the production of doubled haploid lines using in vitro culture. Among the relevant factors affecting the culture efficiency are Cu(II) or Ag(I) acting, e.g., as cofactors of enzymes. The copper ions are known to positively affect green plant regeneration efficiency. However, the biochemical basis, mainly its role in the generation of in vitro-induced genetic and epigenetic variation and green plant regeneration efficiency, is not well understood. Here, we employed structural equation modeling to evaluate the relationship between de novo DNA methylation affecting the asymmetric context of CHH sequences, the methylation-sensitive Amplified Fragment Length Polymorphism related sequence variation, and the concentration of Cu(II) and Ag(I) ions in induction media, as well as their effect on S-adenosyl-L-methionine perturbations, observed using FTIR spectroscopy, and the green plant regeneration efficiency. Our results allowed the construction of a theory-based model reflecting the biological phenomena associated with green plant regeneration efficiency. Furthermore, it is shown that Cu(II) ions in induction media affect plant regeneration, and by manipulating their concentration, the regeneration efficiency can be altered. Additionally, S-adenosyl-L-methionine is involved in the efficiency of green plant regeneration through methylation of the asymmetric CHH sequence related to de novo methylation. This shows that the Yang cycle may impact the production of green regenerants.

## 1. Introduction

Triticale is an allopolyploid plant that was generated artificially [1] and is reasonably simple for in vitro plant regeneration [2], resulting in doubled haploid (DH) lines that could be utilized for the evaluation of new varieties [3]. Chromosome elimination [4] or microspore embryogenesis (androgenesis) [5] are two methods that could be used to create DH plants. In order to take advantage of androgenesis, isolated microspore cultures [6] or anther cultures [7] are used. Green plant regeneration efficiency (GPRE) is constrained by tissue culture media or genotype in both situations [8]. Furthermore, poor spontaneous chromosome duplication [9,10], ineffective regeneration from androgenic structures [11], and the dominance of albino over green regenerants [12,13] are all factors that limit GPRE, with the latter being the most problematic.

From cytological examinations to genomic studies focused on the plastid and nuclear genes, research pertaining to the origin of albino plants have been conducted [14]. Recent investigation on albino plants has revealed a considerable genotypic influence on molecular variations related to chloroplast differentiation in the formation of green and albino regenerants through isolated microspore cultures [15]. According to Sakamoto [16] and Clément [17], the production of amyloplasts from proplastids is necessary for the acquisition of green regenerants. The lack of proplastids prevents the nascent regenerants from carrying out photosynthesis and, thus, does not allow them to function outside the in vitro culture [18,19,20]. From current research on albinism, which takes into account both gene level and studies on structural changes in plastids during androgenesis, it may be deduced that crosstalk between plastid and nuclear genomes is crucial for the appropriate development and function of the chloroplasts and the cell. Through anterograde and retrograde signaling between the cell nucleus and plastid genomes, both genomes are coordinated. However, there are not many interdisciplinary studies relating different GPRE characteristics (such as genetic, epigenetic, metabolomic, and transcriptomic) in cereals.

Ion components constituting the induction media (IM) have the potential to have a significant impact on GPRE [21]. The copper, zinc, and silver ions were notable among the IM constituents. When employed at the proper concentrations, each of these ions had a favorable impact on GPRE [22,23,24,25]. The biochemistry of GPRE and its relationship to de novo methylation, sequence variation, and metal ion concentrations in the IM are, however, poorly understood.

Copper ions (Cu(II)) are elements of proteins such as plastocyanin (act as a cofactor) [26] and cytochrome *c* oxidase [27] involved in electron transport in chloroplast and mitochondria, respectively, and copper/zinc superoxide dismutase (Cu/Zn-SOD) found in the cytosol and chloroplast of plants [28]. By changing the concentrations of Cu(II), it is possible to change the photochemical activity of cells [29,30] or the way enzymes work [31]. Copper ion deficit due to poor ATP generation caused by cytochrome *c* oxidase malfunction may have an impact on S-adenosyl-L-methionine (SAM) production [32,33,34]. Involved in cellular methylation processes like cytosine methylation, SAM is produced in the Yang cycle [35,36]. As suggested by studies on barley [37], SAM may be essential for controlling gene expression [38,39] in order to linking varying parts of GPRE. However, it is not clear if SAM is also involved in GPRE in triticale or if the various components that contribute to the phenomena interact similarly in different species.

If a specially created biological system based on a (epi)genetically uniform progeny of a DH plant is employed as a source of tissue explants for the acquisition of regenerants, research on the genetic and epigenetic components of GPRE might be conducted. When such a system is used, the so-called pre-existing variation [40,41] that may affect the overall variance assessed between regenerants is practically eliminated [42].

Additionally, a molecular marker system that can detect and measure (epi)genetic variation is required. The preferred techniques are Methylation-Sensitive Amplified Polymorphism (MSAP) [43] and methylation-sensitive Amplified Fragment Length Polymorphism (metAFLP) [44]. To quantify sequence variation (SV), DNA demethylation (DMV), and de novo methylation (DNMV) simultaneously, only the metAFLP is available. As shown in some cereals [45,46], it is likewise capable of measuring symmetric (CG, CHG) and asymmetric (CHH) (H is A, C, or T) DNA sequence contexts, where cytosine can be methylated. When only methylation changes are considered, then MSAP could be utilized [44].

A quick, label-free, non-destructive method for determining the metabolome profile of biological samples is infrared spectroscopy, which enables the assessment of a plant’s physiological state, as well as how well it responds to various environmental factors [47,48,49,50,51,52]. Typical plant tissue spectra exhibit several bands caused by the superimposition of a variety of chemical components. Numerous statistical and/or chemometric techniques have been used to extract more detailed information from the spectra, including supervised and unsupervised techniques [53,54,55,56,57,58,59]. Regression analysis and calibration standards are combined in this technique to enable the measurement of specific elements in the material under study [60,61,62]. As shown in experiments on barley by Bednarek [44], it might also be used to quantify a variety of products of metabolic cycles of pathways including SAM.

It is difficult to identify the most critical predictors of any phenomena without sufficient statistical tools and numerous data derived through many methods. This is especially the case when testing a hypothesis describing relationships between varying aspects of a phenomenon. Recently, moderation [43], mediation [46], and structural equation modeling (SEM) statistical methods derived from psychological studies were applied to studies of anther tissue cultures in barley [44]. Furthermore, combining molecular and biochemical (i.e., metabolomic) information and applying moderation, mediation, or SEM to the situation may result in theory-based models that combine various aspects of GPRE into a single framework.

We hypothesized that producing triticale DH regenerants via anther cultures following varying conditions (including the IM ingredients concentrations and time of cultures) from a single uniform plant that is the progeny of a doubled haploid would distinctly alter DNA methylation, disrupt biochemical cycles (i.e., Krebs, Yang), and adjust the metabolome (like SAM) in a way that would have an impact on GPRE. The datasets can be placed in a statistical context to demonstrate their relationships and provide an explanation for GPRE based on biological processes.

The purpose of the study was to evaluate the interactions between de novo DNA methylation affecting the asymmetric sequence context CHH, respective sequence variation assessed with the metAFLP technique [45], Cu(II) and Ag(I) ion concentrations in the IM, and their effects on SAM disturbances revealed via Fourier Transfer Infrared (FTIR) spectroscopy affecting GPRE, using structural equation modeling.

## 2. Materials and Methods

The studies were performed on donor plants and regenerants of hexaploid triticale cultivar T28/2 obtained from a cross between cv. Presto × cv. Mungis. The seeds were provided courtesy of Sylwia Oleszczuk (Plant Breeding and Acclimatization Institute-National Research Institute, Radzików, Poland). Donor plants and regenerants were derived as described elsewhere [63]. Donor plants were the generative progeny of regenerants derived by androgenesis. In addition, donor plants were the source of explants (anthers) for obtaining regenerants. Supplementing induction media with Cu(II) and silver (Ag(I)) ions were tested, and the effect of anther incubation time on induction media was studied. Eight (A-H) different triticale regeneration conditions were tested in the experiment.

The metAFLP technique was used to generate the molecular data [45,64]. The quantification of in vitro-induced variation and its components, SV, DNMV, and DMV, including symmetric (CG and CHG) and asymmetric (CHH) sequence contexts, was performed as described earlier [45].

For the infrared spectroscopy, we used plant material obtained earlier [46]. It consisted of 37 regenerants encompassing eight (A-H) different in vitro conditions. In brief, the infrared spectra were collected from lyophilized and homogenized (ball milled, MM 400, Retsch, Haan, Germany) plant tissues by means of the iZ10 spectrometer (Thermo Fisher Scientific, Waltham, MA, USA) in the Attenuated Total Reflectance (ATR) mode equipped with a deuterated triglycine sulfate (DTGS). The sixty-four scans captured at the 4 cm^−1^ resolution were averaged, ATR and baseline corrected using OMNIC (v.9.0, (Thermo Fisher Scientific, Waltham, MA, USA) software. Following that, the data were Savitzky–Golay smoothed (to reduce high-frequency noise in a signal) and normalized to the unit area (1800–900 cm^−1^) using the hyperSpec package [65] on the R platform [66]. Based on the spectra, absorbances were summarized within 10 cm^−1^ wide intervals across the spectra range from 600 to 3800 cm^−1^ using the hyperSpec package. Furthermore, the SAM reference sample (#A4377, Sigma-Aldrich, St. Louis, MO, USA) was analyzed using the same equipment and procedure as the one mentioned above.

The SEM was performed using Amos™ 20 [67] computer software under iIBM SPSS^®^ [68]. The maximum likelihood (ML) estimation with the Levenberg–Marquardt iteration method [69,70,71] was used to optimize the parameters of proposed models.

## 3. Results

Pachota [63] previously described morphologically uniform donor plants and their regenerants in terms of plant development, leaf shape, color, width, tillering mechanism, and spike number. Orłowska [46] published the quantitative metAFLP features relating to DNA de novo methylation and sequence variation in the asymmetric sequence contexts (CHH). The same is true for the estimation of GPRE [45]. So, Table 1 only shows the GPRE, metAFLP quantitative features and in vitro tissue culture conditions for simplicity.

**Figure 1:**
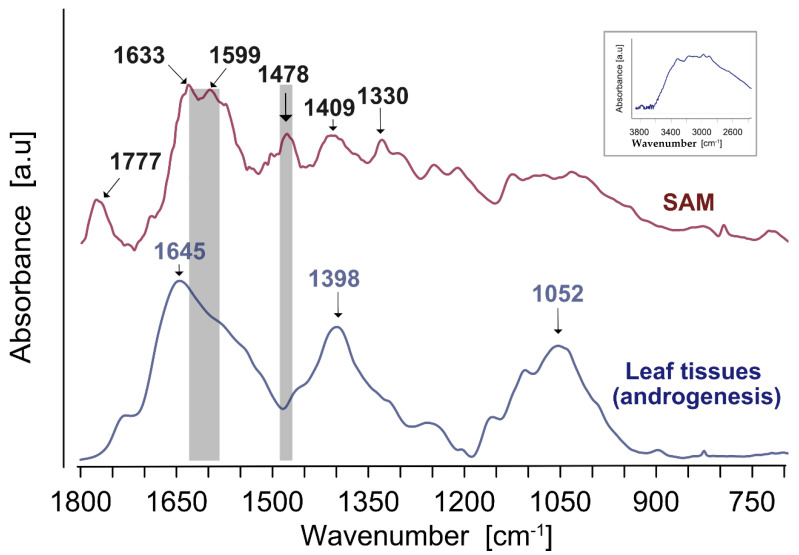
The infrared spectrum of S-adenosyl-L-methionine (upper) and the mean spectrum of leaf tissues (lower). The latter was averaged over all experimental treatments. The major bands are indicated with arrows. The fitted spectra regions in the structural equation modeling are shaded in grey. The inset presents the high-frequency spectral region of SAM.

The infrared spectrum of the SAM compound used as a standard revealed the presence of broadband between 2700 and 3500 cm^−1^ (insert in Figure 1). This absorbance region is attributed mainly to the O-H stretching from ribose and methionine, with only very small local absorbances at 3314 and 3172 cm^−1^, possibly due to the N-H stretching of the amide groups. Additional small local peaks at 2982 and 2909 cm^−1^ are probably derived from the C-H stretching vibrations. Unfortunately, this spectra range did not offer clear high or medium-intensity infrared signals and thus was not used in further analyses as a characteristic of SAM.

The SAM infrared spectra in the region between 1800 and 600 cm^−1^ (Figure 1, upper) showed a band at 1777 cm^−1^, which can be tentatively assigned to C=O stretching, and the double most pronounced peak at around 1600–1630 cm^−1^. The latter may be associated with signals from methionine, namely due to C-C, C-N stretching, the NH_2_ scissoring, and asymmetric stretch of COO- [72,73] as well as from pyrimidine ring vibrations coupled with the NH_2_ scissoring mode of adenosine [74,75]. Other medium bands at 1478 and 1409 cm^−1^ may be attributed to the C-N, C-C stretching, CH_3_ deformation, and NH_2_ bending, while the band at 1330 cm^−1^ is mainly due to the stretch of the C-N, C=N of an adenine ring, and twisting of CH_2_ groups in methionine [76]. Additionally, relatively weaker peaks were distinguished at 1279, 1248, and 1074 cm^−1^, possibly due to the C-C, C-N stretch, and rocking vibrations of NH_2_ deformation [75]. Small absorbances at 821 and 726 cm^−1^ may be attributed to the 5-ring and 6-ring deformations, respectively [76]. Thus, only FTIR wavenumbers assigned to bands allocated to the SAM (around 1330, 1410, 1480, 1580–1630, and 1780 cm^−1^) and present within 1300–1780 cm^−1^ were implemented in further analyses.

Averaged infrared spectra of leaf tissues (Figure 1 lower) showed the most pronounced broadband in the Amide I region, centered at 1645 cm^−1^, possibly due to C-C, C=C, C-N stretching, and NH_2_ deformation. Two complex bands in the fingerprint region were present at 1398 and 1052 cm^−1^. The former could be assigned to vibration modes also attributed to SAM, while the latter was mainly from sugars and structural polysaccharides (the C-C, C-O, and C-C-O stretching) (polysaccharides [77]). The spectra of leaves showed relatively broadband with overlapping absorbances from various chemical compounds in the tissues. Not all FTIR bands detected for SAM were visualized on the leave spectra. Still, they were implemented in the structural equation modeling to identify the regions reflecting SAM vibrations and fit the postulated model. Furthermore, the range of wavenumbers corresponding to absorbances (summarized over 10 cm^−1^ bands) characteristic of SAM was tested (the most relevant signals from SAM are shaded grey in Figure 1).

The postulated model assumed that prominent FTIR bands identified in the SAM spectrum affected de novo DNA methylation (SAM is the cell methylation agent) within different sequence contexts. DNA methylation is influenced by Cu(II) ions, leading to modified methylated cytosines followed by sequence variation. DNMV, SV, and Cu(II) influence GPRE. However, DNMV impacts epigenetic regulation, while Cu(II) affects biochemical pathways, influencing epigenetic mechanisms.

All FTIR bands assigned to SAM within the 1300–1780 cm^−1^ range were tested to fit the postulated model (Table 2). They resulted in at least well-fitting models; however, only the 1590–1630and 1470–1490 cm^−1^ spectral regions and their combination (1470…1630 cm^−1^) gave rise to nearly all significant paths. In contrast, the others failed to identify the significant path toward the DNA methylation variable or sequence variation and did not affect GPRE. Thus, the final SEM model was constructed using only the 1470…1630cm^−1^ spectral region.

The structural equation modeling analysis was based on 37 regenerants representing eight experimental trials (Table 3). A slight deviation from the normal distribution was observed based on skewness and kurtosis values. However, all variables were quantitative and fulfilled the conditions of the Lindeberg–Lévy theorem [78]. Thus, asymptotic convergency with the theoretical normal distribution of the variables is assumed.

Pearson’s linear correlation coefficients (Table 4) show that Cu (II) was positively correlated with GPRE, while a negative correlation was evaluated for CHH_SV. Ag(I) was negatively correlated with CHH_DNMV, whereas anther culture time was positively correlated with CHH_SV. CHH_DNMV was positively correlated with F1630…1470. The other correlations were insignificant.

**Figure 2:**
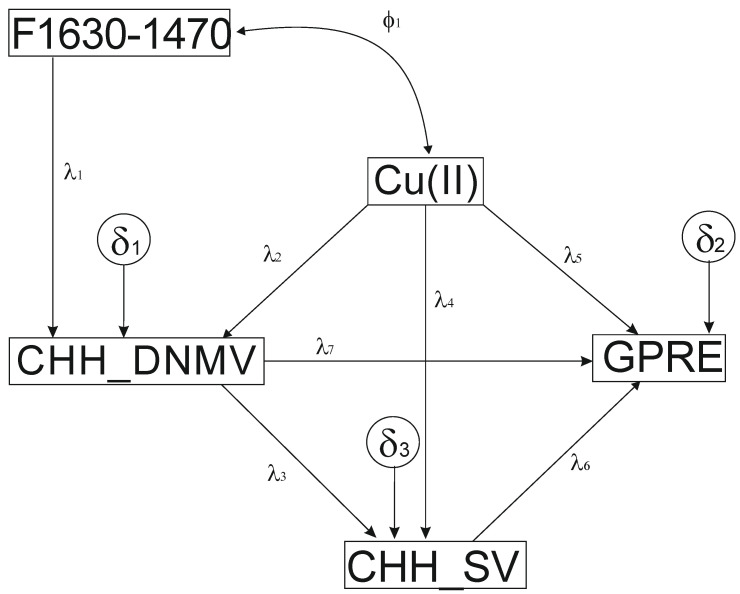
The hypothesized SEM model. Cu(II) ions concentration (μM); GPRE—green plant regeneration efficiency (number of regenerants per 100 plated anthers); the metAFLP quantitative characteristics concerning sequence variation (CHH_SV) and de novo DNA methylation (CHH_DNMV) between donor plant and its regenerants affect asymmetric CHH sequence contexts; The 1630…1470 cm^−1^ wavenumber index represents the FTIR spectrum range assigned to SAM. *λ1–λ7* path coefficients, *δ1–δ3* residuals (experimental errors), and *δ1* a covariate.

The model only uses three endogenous variables (CHH_DNMV, CHH_SV, and GPRE) and two observed variables (Cu (II) and F1630...1470). The relationships had a single covariance (Cu(II) and F1630...1470) and were non-recursive. The model included three residuals (Figure 2).

As not all spectra assigned to SAM resulted in significant models, the final model encompassed the combined FTIR F1630_1590 cm^−1^ and F1490_1470 cm^−1^ spectra, resulting in well-fitting models and the most significant paths (Table 2 and Table 5). Thus, only the combined model is reported in details.

An insignificant chi-squared value indicates a well-fitting model when the sample size is large. However, when the sample size is restricted (as in the case of our data), the test may result in incorrect outcomes [79]. In such a case, using the chi-squared test as the information criterion only [80] is suggested. Thus, additional model descriptive goodness-of-fit measures were used for the model fit evaluation. Nearly all the goodness-of-fit measures were close to the cut-off values [81] or exceeded them. The AGFI and GFI were above 0.95 rigorous cut-off value [82]. The PGFI was around 0.13. The PNFI and PCFI were relatively small, indicating model complexity. Still, the SRMR parameter was below 0.05, demonstrating excellent fitting of the hypothesized model to the data. A similar value was evaluated for the RMR. Additionally, TLI and CFI were close to 1, and RMSEA was less than 0.05, which shows that the model fits the experimental data very well (Table 2).

All the paths’ (*b*) coefficients of the hypothesized model were significant (Table 5) except for the CHH_SV→GPRE. The effect of Cu(II) on GPRE (*β*) was the highest and most positive. The effect of Cu(II) on CHH_SV was the next one, but negative, followed by the CHH_DNMV→CHH_SV, which was positive. The impact of the F1630…1470 variable on CHH_DNMV was also significant and positive, whereas CHH_DNMV negatively affected GPRE. The CHH_DNMV→Cu(II) was also positive and the lowest according to absolute values. Cu (II) and F1630-1470 had no significant covariance.

The hypothesized model includes direct, indirect, and total effects (Table 6). The GPRE variable showed the greatest dependence on Cu (II) (direct: *β* = 0.9863 and total: *β* = 0.9863 with negative indirect: *β* = −0.1712 effects). The CHH_SV depended negatively on Cu (II) (*β* = −0.518 (direct); *β* = −0.3972 (total) and indirect *β* = 0.1208 effects)) but positively on CHH_DNMV (*β* = 0.4131 direct and total effects). The CHH_DNMV was mostly affected by F1630…1470 via direct and total effects (*β* = 0.3669). Finally, the direct effect of Cu (II) (*β* = 0.2924) on CHH_DNMV was observed.

The model did not account for the time of another culture (T) and the amount of Ag(I). Additionally, any other sequence context (CG or CHG), including SV, DMV, or DNMV, did not work when added to the proposed model.

## 4. Discussion

Infrared spectroscopy, particularly the FTIR in ATR mode, is a convenient technique widely used for comparative studies of the biochemical profiles of biological materials. It is a fast, label-free, non-destructive, high-throughput approach successfully applied in taxonomic classification, searching for developmental modifications of phenotype, response to growth conditions, and environmental stresses [53,83,84,85,86,87]. It proved its credit in our previous studies on barley leaves [37] from tissue culture to show the involvement of the Yang cycle in response to ionic stress.

Here, we attempted to link the metAFLP quantitative characteristics of tissue culture-induced variation (TCIV), including DNA methylation pattern, sequence variation evaluated in the triticale regenerants, varying times of anther cultures, the S-adenosyl-L-methionine synthesis fluctuations, and GPRE related to variable Cu(II) and Ag(I) ion concentrations in the IM, by employing structural equation modeling to test the hypothesis that all the data could be combined into a single statistical model of GPRE. It is important to note that except for the FTIR data concerning the SAM spectra, all of the other results have already been discussed in our previous papers [46,63]. So, to keep the presentation simple, we focused on the FTIR data and the SEM model.

Generally, the plant leaves showed complex spectra, where bands from multiple compounds, including those from SAM, overlapped in the most informative region between 1700 and 900 cm^−1^. Thus, identifying a single spectral region characteristic of SAM was hardly possible. We have analyzed the SAM reference compound to identify the most prominent FTIR spectra ranges. We used as the inputs to the model only absorbances for the band regions that corresponded to the most prominent bands of the reference spectrum of SAM. The contribution of each of them was subsequently evaluated by analyzing the model performance. The presented approach was used by us in earlier studies on the input of glutathione in GPRE [81]. However, in those cases, a less complicated banding pattern was observed with just one feature of the GSH spectrum range, which made it easier to choose the right FTIR region.

Out of the five putative regions (Table 2), three of them failed to be informative. The region between 1310 and 1300 cm^−1^ that may be justified by a relatively strong signal from the adenine (the C-C, C=N stretching vibrations, and twisting of CH_2_ groups in methionine) failed to build an SEM model with all informative paths (not shown). As indicated by some goodness-of-fit statistics, the band around 1410 cm^−1^ from the SAM spectrum did not match the model. Overlapping the SAM band vibrations with a strong band centered at 1398 cm^−1^ from multiple tissue compounds could explain the lack of informativeness of the region. Remarkably, this infrared spectrum range may be assigned the O-H in-plane bending mode from phenols and the symmetric stretching of COO- from carboxylic acids. Furthermore, the 1770–1780 cm^−1^ region related to C=O stretching failed to be helpful in SEM model construction due to the lack of significance of some paths rather than problems with reasonably good goodness-of-fit statistics. Again, we speculated that overlapping signals masked the vibrations from SAM’s C=O bands in leaf materials. Lastly, the carbohydrate spectral fingerprint between 1200 and 900 cm^−1^, which shows the presence of simple sugars and cell wall polysaccharides, was not considered because it was thought to hide weak SAM signals.

On the other hand, the 1470–1490 cm^−1^ and 1570–1630 cm^−1^ FTIR spectra ranges allowed the proposed models to have both model-fit indices as expected for excellent model fitting and with significant paths, except for one from SV_CHH to GPRE, which was close to significance (*p* = 0.0506) but turned out to be important in other studies [46]. As the path stabilized the model and was significant in earlier cases [46], we also decided to retain it in the current study. The 1630 to 1590 cm^−1^ range was related to the adenosine and methionine compounds of SAM. Notably, it corresponds to the C-C and C-N stretches in pyrimidine and to the scissoring vibrations of the NH_2_ group present in adenine and methionine [72,73,75] band vibrations. This region does not correspond with maximum absorbance leaf spectra and is located on the shoulder of the Amide I band. The amide I band arises from various vibration modes, including C-C, C=O, C=C, C-N stretch, NH_2_ scissoring, N-H deformation of amino acids, peptides, and asymmetric stretching of COO- in organic acids and pectins. Its location on the shoulder possibly makes it visible in the model since the region near Amide I peak in the leaf spectra (1630–1660 cm^−1^) showed large variability due to the contribution of other more abundant compounds in leaf tissues. Remarkably, the spectral regions outside of this range could interfere with the contributions from amino acids, organic acids, and proteins, the variability of which could mask the effect of SAM. The 1470–1490 cm^−1^ FTIR spectrum, which reflects C-N, C-C stretching, CH_3_ deformation, and NH_2_ bending stretching [76], was helpful in the proposed model. As the vibrations were typical for SAM bands (see the FTIR graph of SAM’s reference compound), we combined them into a single variable utilized for the model construction. It is worth mentioning that the whole FTIR spectrum range was tested in the model; however, only some regions associated with band vibrations present in SAM resulted in excellent fitting SEM models. So, this proves our theory that FTIR spectra show how SAM affects de novo DNA methylation, thus confirming our hypothesis that FTIR spectra reflect SAM-influencing de novo DNA methylation, which is congruent with the knowledge linking the Yang biochemical cycles.

Despite a limited number of regenerants used to construct the structural equation model, the fit indices confirmed that the model with combined FTIR spectra well fitted the data. Even though some deviation from the variables’ normality (indicated by skewness and kurtosis), the deviations were acceptable for further analysis. We have also identified significant correlations between variables, showing that they may be used for model construction. All the paths (maybe except for CHH_SV→GPRE) were significant. As Cu(II) and Ag(I) were used as ingredients added to the IM, the two variables should be treated as exogenous. Usually, it is assumed that such variables impact other variables. However, it could be possible that another non-experimental variables could also act exogenously. On such an occasion, the variable is controlled by an unknown exogenous variable that was not assumed. This is possibly the case in our model where the F1630…1470 cm^−1^ variable reflecting some band vibrations from SAM was treated as exogenous. There were no evident relationships between the two exogenous variables (confirmed by the lack of correlation). Furthermore, SAM seems not to influence GPRE. It is possible that the model needs to be tested with intermediate compounds such as glutathione to show the relationships.

Interestingly, we did not include Ag(I) in the model, which is somewhat surprising given that Ag(I) supplementation in tissue culture media appears to improve plant regeneration efficiency [24,88]. In addition, Orłowska [89] demonstrated the role of Ag(I) in the triticale model. However, the path indicating its influence on GPRE was weak (although significant), as indicated by the standardized *β* value [89]. It is not surprising that by having a limited number of regenerants and a relatively weak path, only those with substantial effects could be retained in models with some other variables. Thus, the Ag(I) variable was not implemented in the current SEM model. In the same way, the time of anther cultures was not included in the model, even though time affected genetic stability [90] and was included in mediation analyses of the comparable relationship in barley [91]. The insignificance of time of anther culture may indicate that it is less important than the other factors and that a larger sample size is needed to verify its impact on GPRE or TCIV characteristics.

The presented model shows the central role of Cu(II) as it impacts de novo DNA methylation and sequence variation of the CHH asymmetric sequence contexts and strongly influences the GPRE. Interestingly, we failed to implement other sequence contexts (CG, CHG) and DNA demethylation in the model. The explanation for that may be linked to the CHH context. It is the least represented methylation type in the cell [92], thus its fluctuations might be easily detected, while the other contexts’ changes are averaged. Another possibility is that CHH de novo DNA methylation differs slightly from that of CG [93] and CHG [94,95]. Chromomethylase 2 (CMT2) [96] and DOMAINS REARRANGED METHYLTRANSFERASE 1/2 (DRM1/2) must establish de novo methylation in the CHH sequence in each generation via the RNA-directed DNA methylation (RdDM) pathway [97]. Whatever the reason, our model demonstrates that SAM influences the CHH sequence context. This is clearly in agreement with the role of SAM in the cell, at least in the case of that sequence context.

Our model is congruent with the putative oxidative stress mechanism that may lead to mutations via the modification of methylated cytosines [98,99] and demonstrates that de novo DNA methylation may participate in GPRE. However, Cu(II) is the most critical factor impacting GPRE. Given the roles of CHH_DNMV and that Cu(II) acts as a cofactor in many biochemical pathways, this could affect the epigenetics that allow GPRE to function. In this context, the role of glutathione should be tested. If the compound changes GPRE, this would be strong evidence that epigenetics plays a role in GPRE.

Surprisingly, SV within the CHH context did not influence GPRE. Sequence variation is one of the causes of somaclonal variation, according to the data [100,101,102,103]. The discrepancy could be explained by the tiny abundance of the CHH methylated sequence in the genome compared to the symmetric contexts. If so, then, as could be expected, our model failed to detect minor effects. Alternatively, a limited sample size might have influenced the statistics, limiting the results. In this context, the presented model should be treated cautiously, demonstrating a putative way of analyzing complex phenomena affecting in vitro tissue culture plant regeneration. However, the model makes it clear that changing the components of the medium can improve GPRE and explains why it is important to understand GPRE in triticale.

## 5. Conclusions

Our results showed that combining metAFLP, biochemical, and absorbance data from infrared spectroscopy evaluated on regenerants derived using a specially designed tissue culture model, limiting pre-existing variation and statistical approaches such as SEM, makes it possible to construct a theory-based model reflecting biological phenomena related to GPRE and demonstrating relationships between variables. We have demonstrated that Cu(II) ions in the IM are the critical factors affecting GPRE. By manipulating their concentrations, it is possible to change the balance of green plant regeneration efficiency. Furthermore, we have shown that SAM is also involved in GPRE indirectly via methylation of the CHH asymmetric sequence contexts via de novo methylation, indicating the involvement of the Yang cycle in GPRE. Our model may miss a significant variable that we did not assume. The putative candidate factor may be glutathione due to its positive effect on microspore embryogenesis in rye [104], the number of embryo-like structures in triticale [105], or embryogenic development in Arabidopsis [106]. Finally, all of these features may influence green plant regeneration efficiency.

## Figures and Tables

**Table 1:** Tissue culture conditions used for plant regeneration by trials A-H, metAFLP quantitative characteristics, the IR spectra region and green plant regeneration efficiency.

Trial	In vitro Anther Culture Conditions *			metAFLP Quantitative Characteristics (%) *		IR Spectra Region, cm^−1^						GPRE *
	Cu	Ag	T	CHH_SV	CHH_DNMV	1300–1310	1390–1410	1470–1490	1570–1630	1770–1780	1470…1630	
A	0.1	10	42	8.66	0.37	0.185803	1.26907	0.42455	2.98543	0.002389	3.40998	0.87
	0.1	10	42	8.66	0.37	0.183574	1.13292	0.43707	3.24874	0.001217	3.68581	0.87
	0.1	10	42	8.52	0.36	0.191639	1.04731	0.53558	3.71659	0.001245	4.25217	0.87
B	0.1	60	49	8.64	0.37	0.178196	1.2209	0.38154	2.85966	0.001426	3.24120	1.52
	0.1	60	49	8.64	0.37	0.254396	1.74913	0.48147	3.51083	0.001349	3.99229	1.52
	0.1	60	49	8.79	0.37	0.227685	1.43375	0.47772	3.48294	0.000922	3.96066	1.52
	0.1	60	49	8.79	0.37	0.175904	1.25097	0.37122	2.89457	0.000428	3.26579	1.52
	0.1	60	49	8.79	0.56	0.202656	0.96207	0.48410	3.35658	0.00006	3.84068	1.52
C	5	60	42	8.76	0.75	0.225474	1.74601	0.48503	3.83905	0.001269	4.32409	0.71
	5	60	42	8.79	0.56	0.190203	0.95596	0.48507	3.77714	0.002415	4.26221	0.71
	5	60	42	8.64	0.55	0.186824	1.69150	0.41648	2.93397	0.003896	3.35044	0.71
D	5	0	49	8.64	0.55	0.219423	1.09671	0.50444	3.83734	0.00393	4.34178	2.38
	5	0	49	8.64	0.55	0.21104	1.07641	0.51469	3.70682	0.002942	4.22150	2.38
	5	0	49	8.76	0.75	0.169463	0.85519	0.48458	3.48811	0.001751	3.97269	2.38
	5	0	49	8.76	0.75	0.233781	1.04895	0.58042	4.02282	0.002706	4.60325	2.38
	5	0	49	8.76	0.75	0.215361	1.54017	0.51606	3.50145	0.002085	4.01752	2.38
	5	0	49	8.76	0.75	0.183114	1.07270	0.49534	3.49364	0.002132	3.98898	2.38
	5	0	49	8.76	0.75	0.210347	1.47711	0.52601	3.73455	-0.000688	4.26056	2.38
	5	0	49	8.76	0.75	0.245707	1.15563	0.57322	4.04161	0.000517	4.61482	2.38
	5	0	49	8.76	0.75	0.214937	1.80068	0.47137	3.71232	0.001001	4.18367	2.38
	5	0	49	8.91	0.76	0.238564	1.38936	0.51576	4.03064	0.001575	4.54641	2.38
E	5	10	35	8.63	0.73	0.250745	1.47001	0.55275	3.68871	0.000681	4.24147	1.17
	5	10	35	8.63	0.73	0.200508	1.00326	0.42343	3.09758	0.001475	3.52101	1.17
	5	10	35	8.48	0.72	0.211908	0.99925	0.45294	3.30125	0.002535	3.75418	1.17
	5	10	35	8.48	0.72	0.225066	1.02850	0.53925	3.54277	0.000399	4.08202	1.17
	5	10	35	8.5	0.54	0.273647	1.55344	0.54718	3.63317	0.00004	4.18035	1.17
F	10	10	49	8.48	0.54	0.182705	1.40998	0.38534	2.92596	0.000861	3.31130	3.79
	10	10	49	8.65	0.55	0.185444	1.32401	0.38477	2.95017	0.000606	3.33494	3.79
	10	10	49	8.65	0.55	0.188182	1.04185	0.43797	3.20739	0.000536	3.64536	3.79
G	10	60	35	8.62	0.56	0.265116	1.81778	0.56006	3.96663	0.002101	4.52669	4.24
	10	60	35	8.49	0.55	0.252854	1.26877	0.58166	3.83262	0.002195	4.41428	4.24
	10	60	35	8.49	0.55	0.253788	1.05274	0.61500	4.03725	0.004113	4.65225	4.24
	10	60	35	8.65	0.55	0.251024	1.68097	0.48234	3.44185	0.00196	3.92419	4.24
H	10	0	42	8.49	0.55	0.221811	1.06542	0.53357	3.73425	0.000346	4.26782	6.06
	10	0	42	8.49	0.55	0.198332	0.97900	0.46960	3.39556	0.001703	3.86516	6.06
	10	0	42	8.65	0.55	0.188945	1.448	0.29188	2.36092	0.003409	2.65280	6.06
	10	0	42	8.65	0.55	0.197657	0.92487	0.46271	3.40941	0.001247	3.87212	6.06

* Data obtained previously as mentioned in the “Material and methods” section; the metAFLP quantitative characteristics concerning sequence variation (CHH_SV) and de novo DNA methylation (CHH_DNMV) between donor plant and its regenerants affect asymmetric CHH sequence contexts; GPRE—green plant regeneration efficiency (number of regenerants per 100 plated anthers on induction medium); IR-infrared.

**Table 2:** The arrangement of SEM goodness-of-fit statistics implementing five FTIR spectra ranges related to SAM.

Fit Indices	FTIR Spectra Intervals (Wavenumbers, cm^−1^)					
	1300–1310	1390–1410	1470–1490	1580–1630	1770–1780	1580–1630; 1470–1490 = 1470…1630
*χ*2	1.2544	3.7445	2.2677	0.0191	0.7437	0.1095
*p*	0.5341	0.1538	0.3218	0.99905	0.6892	0.9467
*df*	2	2	2	2	2	2
RMR	0.0006	0.0121	0.0007	0.0013	0.0000	0.0020
GFI	0.9865	0.9620	0.9762	0.9948	0.9919	0.9988
AGFI	0.8987	0.7149	0.8212	0.9984	0.9392	0.9909
PGFI	0.1315	0.1283	0.1302	0.1333	0.1323	0.1332
NFI	0.9819	0.9962	0.9688	0.9997	0.9891	0.9985
RFI	0.9094	0.7311	0.8439	0.9987	0.9954	0.9924
IFI	1.0111	0.9742	0.9962	1.0283	1.0190	1.0027
TLI	1.0629	0.8537	0.9786	1.1596	1.1081	1.1525
CFI	1	0.9707	0.9957	1	1	1
PNFI	0.1964	0.1892	0.1938	0.1944	0.1978	0.1997
PCFI	0.2	0.1941	0.1991	0.2	0.2	0.2
RMSEA	0	0.1557	0.0610	0	0	0
SRMR	0.0392	0.0478	0.0502	0.0045	0.0182	0.0112

**Table 3:** Descriptive statistics of the variables present in the postulated model including prominent FTIR spectral bands of SEM.

Variable	Min	Max	Mean	SE	SD	Variance	Skewness	Kurtosis
[Cu (II)]	0	10	5.43	0.588	3.576	12.787	−0.102	−1.008
[Ag (I)]	0	60	22.43	4.391	26.709	713.363	0.704	−1.480
[Time (days)]	35	49	43.70	0.955	5.811	33.770	−0.495	−1.371
[CHH_DNMV]	0.36	0.76	0.5838	0.02252	0.13698	0.019	−0.194	−1.054
[CHH_SV]	8.48	8.91	8.6546	0.01884	0.11462	0.013	−0.079	−0.754
[1630…1470]	2.6528	4.6523	3.9617	0.0762	0.4635	0.215	−0.709	0.278
[GPRE]	0.71	6.06	2.5563	0.2727	1.6589	2.752	0.932	−0.122

**Table 4:** Pearson’s linear correlation coefficients for analyzed variables.

Variable	[Cu (II)]	[Ag (I)]	[Time (days)]	[CHH_ DNMV]	[CHH_SV]	[1630…1470]	[GPRE]
[Cu (II)]	1						
[Ag (I)]	−0.181	1					
[Time (days)]	−0.312	−0.203	1				
[CHH_DNMV]	0.320	−0.426 **	−0.028	1			
[CHH_SV]	−0.386 *	0.050	0.607 **	0.247	1		
[1630…1470]	0.075	−0.020	−0.116	0.389 *	0.080	1	
[GPRE]	0.807 **	−0.201	−0.061	0.005	−0.297	−0.052	1

* Correlation is significant at the 0.05 level (2-tailed); **. Correlation is significant at the 0.01 level (2-tailed).

**Table 5:** Path coefficients, variances and covariances for the analyzed model.

Parameter	Effect			Estimate (*b*)	SE	Test Statistic	Standardized (*β*)
*Path coefficients*							
λ1	[1630…1470]	→	[CHH_DNMV]	0.1084	0.0432	2.5118 *	0.3669
λ2	[Cu(II)]	→	[CHH_DNMV]	0.0112	0.0056	2.0019 **	0.2924
λ3	[CHH_DNMV]	→	[CHH_SV]	0.3457	0.123	2.8115 **	0.4131
λ4	[Cu(II)]	→	[CHH_SV]	−0.0166	0.0047	−3.5252 ***	−0.518
λ5	[Cu(II)]	→	[GPRE]	0.4576	0.0479	9.552 ***	0.9863
λ6	[CHH_SV]	→	[GPRE]	2.4767	1.4615	1.6947	0.1711
λ7	[CHH_DNMV]	→	[GPRE]	−4.2763	1.1907	−3.5914 ***	−0.3531
*Covariance*							
*φ*_1_	Cu(II)	←→	[F1630…1470]	0.1206	0.2695	0.4474	
*Variances*							
δ1				0.0139	0.0033	4.24264 ***	
δ2				0.0089	0.0021	4.24264 ***	
δ3				0.6859	0.1617	4.24264 ***	
[Cu(II)]				12.4414	2.9325	4.24264 ***	
[1630…1470]				0.209	0.0493	4.24264 ***	

*—significant at *p* ≤ 0.05; **—significant at *p* ≤ 0.01; ***—significant at *p* ≤ 0.001

**Table 6:** Direct, indirect and total effects for the analyzed model.

Effect			Estimates (*b*)			Standardized Estimates (*β*)		
			Direct	Indirect	Total	Direct	Indirect	Total
[GPRE]								
[1630…1470]	→	[GPRE]	-	−0.3709	−0.3709	-	−0.1036	−0.1036
[Cu(II)]	→	[GPRE]	0.4576	−0.0794	0.3781	0.9863	−0.1712	0.8151
[CHH_ DNMV]	→	[GPRE]	−4.2763	0.8562	−3.4201	−0.3531	0.0707	−0.2824
[CHH_SV]	→	[GPRE]	2.4767	-	2.4767	0.1711	-	0.1711
[CHH_SV]								
[1630…1470]	→	[CHH_SV]	-	0.0375	0.0375	-	0.1516	0.1516
[Cu(II)]	→	[CHH_SV]	−0.0166	0.0039	−0.0127	−0.5180	0.1208	−0.3972
[CHH_ DNMV]	→	[CHH_SV]	0.3457	-	0.3457	0.4131	-	0.4131
[CHH_DNMV]								
[1630…1470]	→	[CHH_DNMV]	0.1084	-	0.1084	0.3669	-	0.3669
[Cu(II)]	→	[CHH_DNMV]	0.0112	-	0.0112	0.2924	-	0.2924

## Data Availability

Not applicable.
